# Comparative Transcriptomics Analysis Reveals Rusty Grain Beetle’s Aggregation Pheromone Biosynthesis Mechanism in Response to Starvation

**DOI:** 10.3390/insects15020137

**Published:** 2024-02-19

**Authors:** Fangfang Zeng, Haixin Jiang, Haoqi Xu, Ruotong Shen, Dianxuan Wang

**Affiliations:** 1 National Grain Industry (Storage Insect Pest Control) Technology Innovation Center, School of Food and Strategic Reserves, Henan University of Technology, Zhengzhou 450001, China; zengfangfang@haut.edu.cn (F.Z.); jianghaixin2022@163.com (H.J.); 17638519102@163.com (H.X.); 18638707670@163.com (R.S.); 2 Grain Storage and Logistics National Engineering Research Center, School of Food and Strategic Reserves, Henan University of Technology, Zhengzhou 450001, China

**Keywords:** *Cryptolestes ferrugineus*, pheromone biosynthesis, FAS pathway, mevalonate (MVA) pathway

## Abstract

**Simple Summary:**

Pheromones, such as sex pheromones, aggregation pheromones, and alarm pheromones, are the basis for insect aggregation, mating, and other behaviors. Compared with the structures of sex pheromones, those of aggregation pheromones are more complex, especially in cucujoid grain beetles, which produce macrocyclic lactones as aggregation pheromones. So far, there is limited research studying the biosynthesis of macrocyclic lactones at the molecular level. Two aggregation pheromone components of the rusty grain beetle, *Cryptolestes ferrugineus*, have been identified to be biosynthesized via the mevalonate (MVA) pathway and fatty acid synthesis (FAS) pathway, with their productions affected by starvation. Therefore, we conducted a comparative transcriptomics analysis of hypothetical pheromone production sites in *C. ferrugineus* under starvation stress. Additionally, significantly enriched metabolic pathways and down-regulated genes directly involved in the biosynthesis and regulation of aggregation pheromones via the MVA and FAS pathways, respectively, were identified, as well as some juvenile hormone (JH)- and insulin-related genes, which are of great significance for elucidating the synthesis and regulatory mechanisms of cucujoid grain beetle pheromones.

**Abstract:**

Pheromones are the basis of insect aggregation, mating, and other behaviors. Cucujoid grain beetles produce macrocyclic lactones as aggregation pheromones, yet research on their biosynthesis at the molecular level remains limited. The rusty grain beetle, *C. ferrugineus*, is an important economic species in China. Although two aggregation pheromone components have been identified, their suspected biosynthesis via the MVA pathway and the FAS pathway lacks molecular elucidation. Previous evidence supports that starvation affects the production of aggregation pheromones. Therefore, we constructed comparative transcriptome libraries of pheromone production sites in *C. ferrugineus* under starvation stress and identified genes related to pheromone biosynthesis and hormone regulation. A total of 2665 genes were significantly differentially expressed, of which 2029 genes were down-regulated in starved beetles. Putative *C. ferrugineus* genes directly involved in pheromone biosynthesis were identified, as well as some genes related to the juvenile hormone (JH) pathway and the insulin pathway, both of which were depressed in the starved beetles, suggesting possible functions in pheromone biosynthesis and regulation. The identification of genes involved in macrolide lactone biosynthesis in vivo holds great significance, aiding in the elucidation of the synthesis and regulatory mechanisms of cucujoid grain beetle pheromones.

## 1. Introduction

Insect pheromones, including moth sex pheromones, beetle aggregation pheromones, ant tracking pheromones, and aphid alarm pheromones, play important roles in insect courtship, foraging, egg laying, alarm, and defense processes [1]. Sex pheromones and aggregation pheromones, produced by female and male insects, respectively, have been widely used as pest control strategies, including monitoring, mass trapping, and disrupting the mating process of pests. This is owing to their species specificity, activity at very low concentrations, and environmental safety. Most previous studies on insect pheromones have focused on identifying pheromone components and understanding the factors affecting pheromone synthesis [2,3,4]. Aggregation pheromones have been identified in more than 300 non-social arthropods [5]. Although the compositions of these pheromones vary widely between families, they are structurally conserved within the same family. Sex pheromones can be classified into two structurally different classes: type I pheromones, which are long, straight-chain aliphatic alcohols, aldehydes, and esters, and type II pheromones, which are epoxides without terminal functional groups. Comparatively, the structures of aggregation pheromones are more complex and diverse than those of sex pheromones, for example, the *bis*-epoxidized heterocyclic structure of the aggregation pheromones in the family Coryphaenidae or the macrolide structure featured in the family Platypodidae [6,7]. Several structurally similar macrolide pheromones have been identified in flat grain beetles. The synthesis and release of these pheromones are influenced by exogenous factors, such as insect maturity, the presence or absence of heterosexuality and food, population density, rhythm, light, temperature, humidity, and gut microbes. Additionally, endogenous substances, such as hormones, the activating neuropeptide PBAN, and androgen appendages play a role [4,8,9,10]. Food intake is a crucial aspect of insect reproduction, requiring coordination between finding food and a mate. Furthermore, insects undergo behavioral and physiological changes as an effective survival strategy during starvation. Behaviors, such as feeding and egg laying, are adjusted based on an insect’s nutritional status [11,12,13,14]. Additionally, sensory responses to sexual and food signals are consistently modulated by food [15]. For instance, compared with satiated *Drosophila melanogaster* fasted *D. melanogaster* exhibited a higher attraction to food odor sources to promote food seeking [16]. They were also observed to change their food preferences in response to starvation [15]. Moreover, in *D. melanogaster* females, insulin signaling partially controls pheromone perception in the antennal lobe (AL) and modulates *cis*-vaccenyl acetate (cVA) attractiveness according to the nutritional status. After 72 h without food, *D. melanogaster* males showed a significant decrease in the number, duration, and occurrence of mating attempts [17]. Male-produced aggregation pheromones attract and group males and females of the same species according to the food source for feeding and mating [18]. However, *Rhodnius prolixus* in a starved state shows repulsion to aggregated pheromones [19]. Similarly, the feeding status significantly affects the quantity of pheromone produced by the tobacco budworm (*Heliothis virescens* (Fabricius)) [20], German cockroaches (*Blattella germanica* (Linnaeus)) [21], and boll weevil (*Anthonomus grandis grandis* Boheman) [22]. In aphids, the weight and quantity of the alarm pheromone EβF are regulated by insulin receptor genes (InsR1/2) and downstream genes (PI3K and Akt), which encode kinases and key enzymes in the glycolysis (HK, A6PFK, and PK) and the isoprenoid (ACSS, HMGR, FPPS1, FPPS2, GGPPS, and DPPS) pathways, and EβF is significantly reduced by nutritional stress [23]. In addition, satiated male mountain pine beetles (*Dendroctonus ponderosae*), produced frontalin ((1R,5R)-1,5-dimethyl-6,8dioxabicyclo [3.2.1]octane) by feeding males via the MVA pathway, acting as an anti-aggregation pheromone signal to halt attacks and prevent the overcrowding of trees [24]. Thus, starvation is often utilized to analyze pheromone biosynthesis pathways in insects [22,24].

Pheromones can be synthesized de novo via the FAS pathway or the MVA pathway and/or by further processing their precursors in food [20]. It is generally accepted that pheromone synthesis is mainly regulated by the activated neuropeptide PBAN in Lepidoptera [8,25], by ecdysteroids in Diptera (houseflies) [7], and by juvenile hormone (JH) III in Coleoptera [26,27]. The regulation of pheromone biosynthesis by endogenous factors can be achieved by affecting the activities of the key enzymes or the expressions of genes at the transcriptional level. For instance, ecdysteroids regulate hydrocarbon properties by affecting the activity of one or more fatty acyl-CoA elongases [7]. On the other hand, JH III juvenile hormone modulates the expression levels of 3-hydroxy-3-methylglutaryl-coenzyme A synthase 1 (HMGS) and 3-hydroxy-3-methylglutaryl-coenzyme A reductase (HMGR) or the enzymatic activity of geranylgeranyl diphosphate synthase (GPPS), thereby controlling the synthesis of pheromones in *Ips paraconfusus* [28]. Consequently, the identification and analysis of genes in the pheromone biosynthesis pathway are a crucial prerequisite for unraveling the molecular mechanisms of pheromone synthesis and regulation.

The rusty grain beetle, *Cryptolestes ferrugineus* (Stephens) (Coleoptera: Laemophloeidae), is among the most prevalent grain pests, infesting stored and marketed cereal grains. *C. ferrugineus* has been reported in more than 110 countries [29]. Previous studies have documented the two main aggregation pheromone components of *C. ferrugineus*: cucujoid I (4,8-dimethyl-(E,E)-4,8-decadienolide) and cucujoid II ((S)-(Z)-3-dodecen-11-olide), which have been isolated from frass [30]. Isotope-labeling experiments have demonstrated that these two compounds are derived from the de novo synthesis of fatty acids and terpenoids, respectively, and are regulated by the feeding state [31]. However, the molecular biosynthesis and regulation mechanisms of cucujoid I and cucujoid II have not been determined yet. Therefore, in this study, we conducted a comparative transcriptome analysis on rusty grain beetles under normal conditions and during starvation stress, identifying significantly differentially expressed pathways and potential key genes involved in the biosynthesis and regulation of pheromones.

## 2. Experiment and Methods

### 2.1. Insect Rearing and Sample Preparation

The *C. ferrugineus* strain was collected from a grain storage depot in Xinxiang, Henan Province, China. The colonies were kept in the laboratory for more than 10 years. Before the experiment, the eggs laid in one day were first collected and then reared with feeding under the conditions of 28 °C and 75% relative humidity until eclosion. Then, the newly emerged adults were separated by sex, and only 7-day-old males were selected for the experiments. After a 24 h starvation period, the guts of the male adult *C. ferrugineus* were collected by dissecting the abdomen of each insect on a wax plate. A total of 100 individuals were used in each replicate, and three biological replicates were conducted. All the collected samples were instantly stored at −80 °C until use.

### 2.2. cDNA Library Construction and Sequencing

The total RNA was isolated from the samples with Trizol reagent (Invitrogen, Carlsbad, CA, USA) according to the manufacturer’s instructions. The RNA concentration and purity were measured with a Nanodrop spectrophotometer (Thermo Fisher Scientific, Waltham, MA, USA). Three micrograms of RNA were used as input material for the sequencing library preparation. RNA libraries were generated using the TruSeq RNA sample preparation kit (Illumina, San Diego, CA, USA). Briefly, the mRNA was first purified from the total RNA using poly-T oligo-attached magnetic beads. Fragmentation was carried out using divalent cations at an elevated temperature in Illumina’s proprietary fragmentation buffer. First-strand cDNA was synthesized using random oligonucleotides and SuperScript II reverse transcriptase. Second-strand cDNA synthesis was subsequently performed using DNA polymerase I and RNase H. The remaining overhangs were converted to blunt ends via exonuclease/polymerase activities, and the enzymes were removed. After the adenylation of the 3′ ends of the DNA fragments, Illumina PE adapter oligonucleotides were ligated to prepare for hybridization. To select the cDNA fragments of the preferred 400–500 bp length, the libraries were further purified using the AMPure XP system (Beckman Coulter, Beverly, CA, USA). DNA fragments with ligated adaptor molecules on both ends were selectively enriched using the Illumina PCR primer cocktail in a 15-cycle PCR reaction. The products were purified (AMPure XP system) and quantified using an Agilent high-sensitivity DNA assay in a Bioanalyzer 2100 system (Agilent Technologies, Santa Clara, CA, USA). The sequencing library was then sequenced on a NovaSeq 6000 platform (Illumina) by Shanghai Personal Biotechnology Co., Ltd., Shanghai, China.

### 2.3. RNA-Seq Analysis

Trinity (version 2.5.1) software (Broad Institute, Cambridge, MA, USA) was used for the de novo assembly of the clean reads. The longest transcript of each gene, also called a unigene, was extracted and annotated using BLASTX and the NCBI non-redundant (NR) protein sequences, the Gene Ontology (GO), the Kyoto Encyclopedia of Genes and Genomes (KEGG), the evolutionary genealogy of genes: Non-supervised Orthologous Groups database (eggNOG), and the SwissProt and the Pfam databases. All the unigenes were then submitted to KEGG PATHWAY under default parameters to group the unigenes into lists of genes participating in the same biological process.

During the differential expression analysis, RSEM (version 1.2.31) was used for the transcript abundance estimation. The RSEM-expected counts were then subjected to DESeq (1.30.0) for the identification of differentially expressed genes (DEGs), with a criterion of |log2 fold change| > 1 and a significant adjusted *p*-value of <0.05.

Next, topGO was utilized to perform GO enrichment analysis on the differential genes, calculate *p*-values via the hypergeometric distribution method (the standard of the significant enrichment is a *p*-value of <0.05), and find the GO term with significantly enriched differential genes to determine the main biological functions performed by differential genes. ClusterProfiler (version 3.4.4) software was used to carry out the enrichment analysis of the KEGG pathway of the differential genes, focusing on the significant enrichment pathway with a *p*-value of <0.05.

Finally, the individual genes related to pheromone biosynthesis were manually selected for further analysis.

### 2.4. Validation of Gene Expression Levels

Gene expression patterns were validated using real-time quantitative PCR (RT-qPCR) on the Bio-Rad according to the previously described protocol [32]. The primers were designed using the Primer-BLAST tool at NCBI (i.e., the primer-designing tool at nih.gov, accessed on 8 February 2024) (Table S1). The specificity and amplification efficiency of the primers used in this study were assessed using standard protocols [22]. The *C. ferrugineus* ARF1 (ADP ribosylation factor) gene was used as an internal reference [32]. The relative expression levels of the selected genes were calculated using the 2^−ΔΔCT^ method [33]. The guts from the starved and fed male beetles were used as the calibrator samples in the RT-qPCR analysis. Three biological replicates were performed for each sample.

### 2.5. Data Analysis

The mean ± SEM (standard error of the mean) of three replicates are presented. And all the statistical analyses were conducted in SPSS (version 20.0) (IBM Corp., Armonk, NY, USA), with differences in the *p*-value of <0.05 being considered as statistically significant.

## 3. Results

### 3.1. Summary of Transcriptome-Sequencing Data

A total of 314,866,174 raw reads were obtained for the six *C. ferrugineus* individuals that were sequenced. After the quality filtration, 295,233,410 clean reads were obtained, representing 19.2 Gb nucleotides, with an average ratio of 93.76% raw reads. The Q20% and Q30% (percentages of nucleotides with pure-read masses greater than 20% and 30%) were 96.78% and 91.67%, respectively (Table 1). After splicing and integration, 1,995,300,442 transcripts were generated, with an average length of 1158.19 bp and an N50 length of 2458 bp. A total of 52,277,232 unigenes were identified, with an average length of 1233.27 bp and an N50 length of 2212 bp (Table S2).

### 3.2. Functional Annotation

The functions of the unigene sequences were evaluated. And a total 16,364 unigenes were annotated with the help of six databases. Among these, 6474 (15.27%) unigenes were annotated in all the databases. The numbers of genes homologous to the sequences in the NR, GO, KEGG, PFAM, eggNOG, and SwissProt databases were 16,364 (38.60%), 12,078 (28.49%), 9570 (22.58%), 11,649 (27.48%), 15,674 (36.98%), and 12,730 (30.03%), respectively (Table S3).

**Figure 1:**
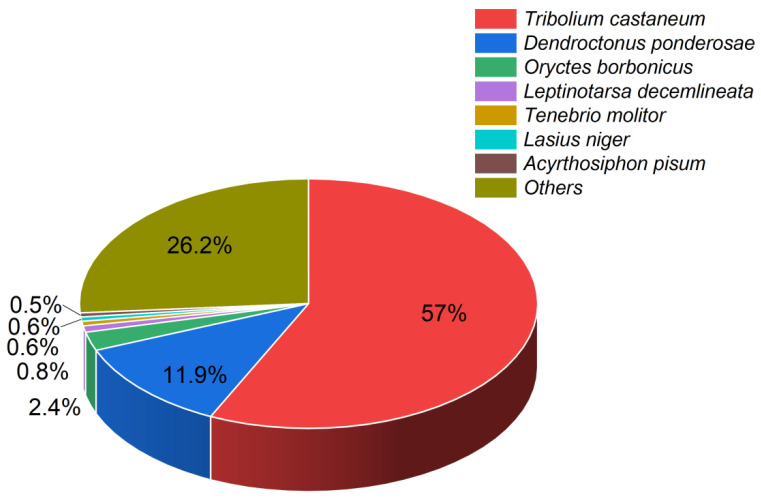
Pie chart illustrating the percentages of identified unigenes with known homologs in other insect species. Each color represents a different species. Percentages indicate proportions of unigenes.

According to the BLAST alignments, the majority of the unigenes mapped to the genomes of species in the order Coleoptera, with predominant mappings to the *Tribolium castaneum* and *Dendroctonus ponderosae* genomes and some to the *Oryctes borbonicus* genome (Figure 1).

**Figure 2:**
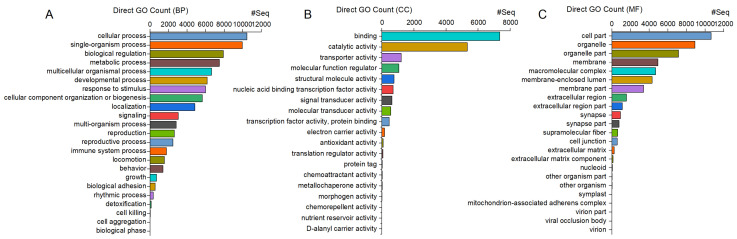
*C. ferrugineus genes* annotated with GO and showing significant enrichment in three main categories: (**A**) biological processes (BPs), (**B**) molecular functions (MFs), and (**C**) cellular components (CCs), with an adjusted *p*-value of <0.05. The *x*-axis indicates the number of genes in each category.

The majority of the 12,078 unigenes annotated with the GO pipeline were enriched in general biological processes (BPs), such as “cellular process” (10,453 unigenes, 87%) and “single-organism process” (9961 unigenes, 82%). Similarly, most of the genes were associated with general cellular components (CCs), such as “cell” (10,651 unigenes, 88%) and “cell part” (10,649 unigenes, 88%), and with molecular functions (MFs) related to “binding” (7355 unigenes, 61%) and “catalytic activity” (5326 unigenes, 44%) (Figure 2).

### 3.3. RNA-Seq Analysis

**Figure 3:**
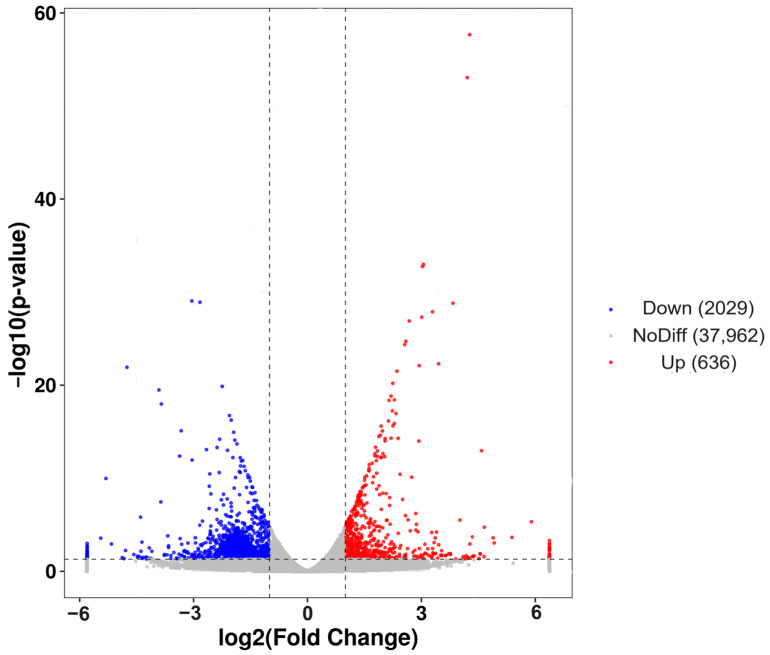
Volcano plot showing the 2479 significantly differentially expressed genes between post-starvation and normally fed *C. ferrugineus* males. Notes: Gray dots represent genes with no difference (NoDiff; 37,962 genes). Blue dots represent down-regulated genes (Down; 2029 genes). Red dots represent up-regulated genes (Up; 636 genes). The gray vertical lines show the −1 and 1 log fold-change thresholds. The *y*-axis shows the negative logarithm (base 10) of the adjusted *p*-value, and the *x*-axis shows the log2 fold change in the gene expression.

A total of 2665 DEGs were identified, with 2029 genes down-regulated in post-starvation males compared with normally fed males, while 636 genes were up-regulated (Figure 3). The overall expression patterns of the DEGs differed between the starvation and non-starvation treatment groups (Figure 3).

**Figure 4:**
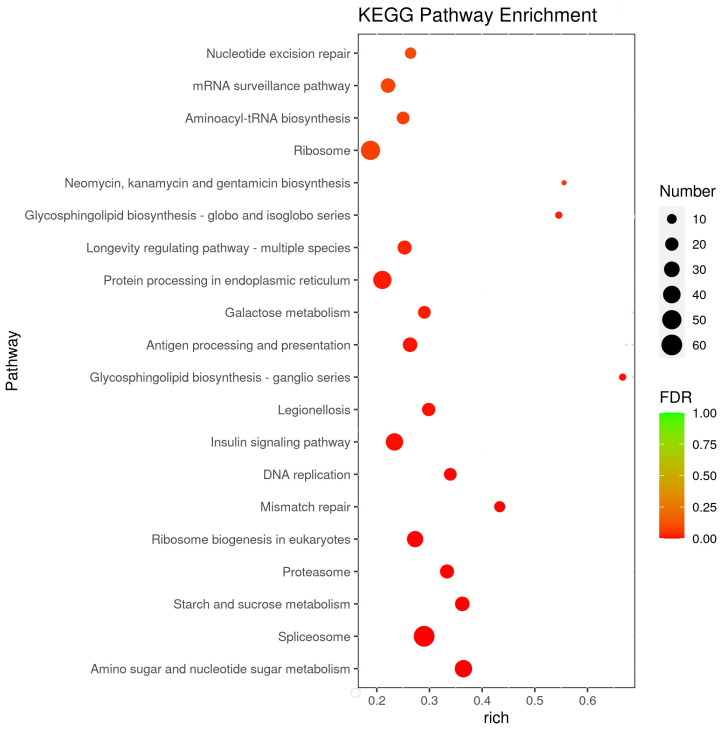
Enriched pathways in KEGG analysis. The *x*-axis represents the gene ratio, which indicates the ratio of the DEGs to all the genes annotated in a particular pathway (*y*-axis). The size of each circle indicates the number of DEGs that are associated with each significant pathway, while the color refers to the significance level of the pathway (green is the highest and red is the lowest), with an adjusted *p*-value of <0.05.

To better understand the main response mechanism in *C. ferrugineus* to starvation, we further studied the pathways with the most significant differences in gene expression (adjusted *p*-value of <0.05) between the starved and normally fed male beetles (Figure 4 and Table S4). The differentially expressed pathways were mainly focused on metabolism and genetic information processing. Although some signaling pathways, including the insulin-signaling pathway and the longevity regulation pathway, were also differentially expressed during starvation.

### 3.4. Differentially Expressed Genes in Putative Pheromone Biosynthesis Pathway

**Figure 5:**
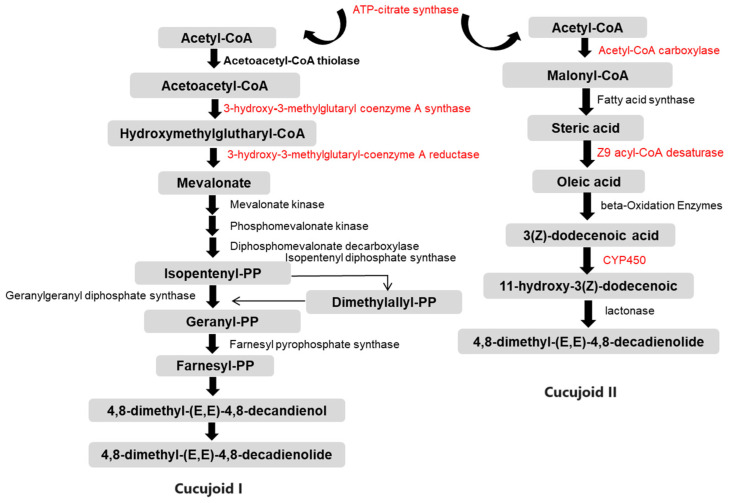
Schematic diagram illustrating the putative pheromone biosynthesis pathways of cucujoid I and cucujoid II in *C. ferrugineus.* Gray indicates the different intermediate molecules of the pathway. Arrows represent different enzyme-catalyzed reactions and indicate the direction of the pathway. Genes that were down-regulated in starved beetles are highlighted in red [31].

Combining the results obtained in this study (Tables S5–S7) with previously obtained knowledge of the intermediates of pheromone synthesis [6], we generate the potential molecular pathways for *C. ferrugineus* aggregation pheromone biosynthesis (Figure 5).

In the mevalonate (MVA) pathway, the expressions of the genes encoding 3-hydroxy-3-methylglutaryl-coenzyme A synthase 1 (HMG-S), 3-hydroxy-3-methylglutaryl-coenzyme A reductase (HMG-R), mevalonate kinase (MK), and ATP-citrate synthase were highly repressed after starvation (Table 2 and Table S6). In the FAS pathway, ten genes showed significantly reduced expression levels, corresponding to annotations for acetyl-CoA carboxylase (ACC), fatty acid synthase (FAS), Z9 acyl-CoA desaturase (DES), fatty acyl-CoA reductase (FAR), alcohol dehydrogenase (ADH), acetyl-CoA binding protein (ACBP), acetyl-CoA acetyltransferase (ATF), and cytochrome P450 (Table 2 and Table S7).

### 3.5. Validation of the Key Genes in the FAS and MVA Pathways

**Figure 6:**
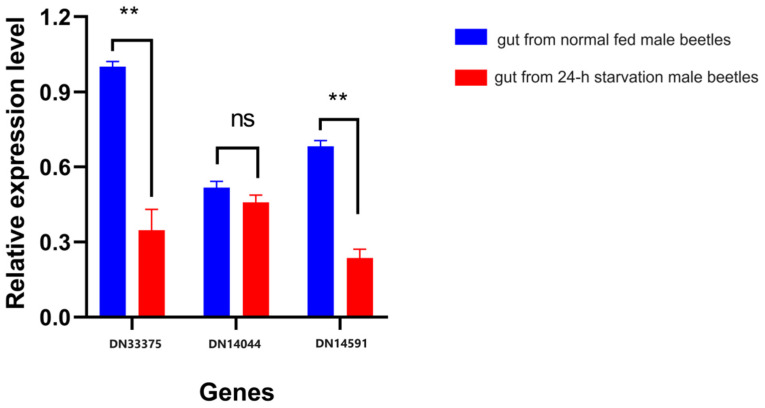
Validation of the gene expression patterns of three key genes in the FAS pathway. Relative expression levels (*y*-axis) of genes DN33375, DN14591, and DN14040 (*x*-axis) in starved (red) and normally fed (blue) *C. ferrugineus* male adults. Asterisks represent significant differences, with a *p*-value of <0.05; “ns” indicates no significant differences between groups.

We validated the expression patterns of three key genes, HMGR, DES, and GPPS, in the FAS and MVA pathways. The up-/down-regulation trends of the three selected genes, N33375, DN14591, and DN14040, corresponding to annotations for HMGR, DES, and GPPS, respectively (Table 2 and Table S6), were consistent with the RNA-seq results, as shown in Figure 6. These findings indicate the high reproducibility and reliability of our transcriptome analysis.

## 4. Discussion

This study reports, for the first time, data regarding the gut transcriptome of *C. ferrugineus*, with a focus on elucidating the biosynthesis of aggregation pheromones at the molecular level. These gene expression data acquired in this project may help to expand the fundamental molecular knowledge of *C. ferrugineus* gene expression and contribute to further molecular research on the biosynthesis and regulation mechanisms of aggregation pheromones in rusty grain beetles.

Many insects use specialized cells in the abdomen or epidermis to produce pheromones [34]. In *C. ferrugineus*, like in bark beetles [35], pheromone substrates were isolated from frass, suggesting a different possible location of pheromone production in the gut. Bark beetles produce isoprenoid aggregation pheromones, such as ipsdienol, ipsenol, and frontalin, in their midgut tissues [36]. With that in mind, we selected the putative DEGs and pathways that exhibited significant differences in expression between the treatment groups, as they may directly contribute to gut pheromone biosynthesis (Table 2 and Figure 4).

The MVA metabolic pathway in insects is initiated by the reductive polymerization of acetyl-CoA, resulting in the production of a variety of isoprenoid compounds. Research on pheromone production and the MVA pathway in insects has been primarily conducted in species such as *Drosophila* and pine beetles, with a focus on elucidating the various steps of the pathway [28]. Our study found that eight genes directly involved in the MVA pathway were down-regulated in starved beetles (Table 2 and Figure 4). Specifically, the genes encoding the enzymes HMG-S, HMG-R, mevalonate kinase (i.e., the enzymes catalyzing the first reaction of the MVA pathway), the enzyme that converts acetyl-CoA to mevalonate 5-PP, and ATP citrate synthase (which converts citrate to acetyl-CoA), which are all required for the initiation of the mevalonate pathway [36], were all down-regulated.

The FAS pathway is one of the most common pathways for pheromone production in insects. It has been widely studied in moths, revealing many indispensable enzymatic reactions, such as desaturation, oxidation, reduction, and acetylation [30]. In *C. ferrugineus*, one of the aggregation pheromone components, cucujoid I, was suggested to be synthesized de novo via the FAS pathway from acetyl-CoA [30]. The biosynthesis of cucujoid II is speculated to occur as follows: Acetyl-CoA is converted to the fatty acid precursor malonyl-CoA by the action of acetyl-CoA carboxylase (ACC) and then acetyl-CoA and malonyl-CoA, acting as substrates, along with NADPH as a reducing agent, are transformed to stearic acid (C18) by fatty acid synthetase (FAS). Subsequently, a double bond is introduced to a specific position in the acyl chain by delta-9-desaturases (DESs) [37,38]. The unsaturated fatty acid undergoes chain shortening through β-oxidation, generating (Z)-3-dodecenoic acid, a specialized chain length intermediate. Following this, the pheromone precursor 11-hydroxy-(Z)-3-dodecenoic acid is obtained via ω-1 hydrolysis [6,7]. The final step involves lactone cyclization, forming macrocyclic lactones as the functional active pheromone components (Figure 5). Ten genes that are related to the fatty acid biosynthesis pathway and pheromone production were found to be highly down-regulated in starved beetles (Table 2), including ACC, FAS, DES, and other genes related to beta-oxidation. In a previous study, ACC was found to be activated by dephosphorylation at positions serine 84 and serine 92 in the PBANR/Ca^2+^/CaN/ACC pathway, ultimately regulated by PBAN [8]. Similarly, the dynamic variation in the sex pheromone titers was disrupted when the expression of the desaturase genes was significantly altered by knocking down the clock gene period (SlitPer) or the knockout of the tyramine receptor 1 gene [38,39]. Furthermore, the FAS gene was highly expressed in pheromone-producing boll weevils (*Anthonomus grandis grandis*) compared with non-pheromone producing weevils, indicating its involvement in pheromone biosynthesis [22].

Cytochrome P450s is a group of widespread metabolic enzymes involved in the oxidation of various of endogenous compounds, such as steroids and hormones, as well as exogenous compounds, including alcohol, drugs, and environmental pollutants [40]. In this study, starvation up-regulated a large number of cytochrome-P450s-related genes, suggesting that they may play a key role in the fight against starvation. Still, some other cytochrome P450s, such as CYP4ac1, which encodes CYP4A, were found to be down-regulated. CYP4A is known to promote the ω-hydroxylation of fatty acids [41], making it one of the least hormonally regulated enzyme targets in *C. ferrugineus* [6].

It has been shown that the biosynthetic pathway of insect pheromones is under the complex control of endocrine hormones [23]. In Coleoptera, pheromone production is thought to be regulated by the hormone JH III [7]. Interestingly, pheromone production was induced in *I. duplicatus* and *I. pini* by JH III during starvation.

In *C. ferrugineus*, pheromones are produced de novo rather than directly using a fatty acid precursor. This suggests that feeding may somehow trigger pheromone production rather than simply alleviate the limitation of the pheromone-precursor’s supply [31]. In our study, one of the JH-related genes (encoding the JH-binding protein) was significantly differentially expressed during starvation. The final step in the MVA pathway is the production of JHs, and, in turn, JHs can regulate the MVA pathway [42]. JH III has been shown to regulate pheromone production in *D*. *melanogaster* and the male midgut of *I. pini* [43,44]. Previously, in *C. ferrugineus*, pheromone production in males fed methoprene-treated oats for 15–21 days was observed to be approximately 2-fold higher than in males fed pentane-treated oats [45]. Our data provide fundamental molecular evidence in support of the JH-regulated hypothesis.

The insulin pathway is believed to be the key in responding to nutrient signaling under starvation conditions in *D. melanogaster*, particularly by up-regulating the expression of genes that are targeted by FOXO, leading to a reduction in insulin signaling [46]. Indeed, novel functions of the insulin-signaling pathway have recently been discovered [23,47]. For instance, the insulin pathway, along with the glycolysis and isoprenoid pathways, has proved to modulate aggregation pheromone biosynthesis in response to starvation stress [23]. Additionally, it regulates the pheromone-mediated avoidance behavior in *Caenorhabditis elegans* [47], a scenario that could also be applied to *C. ferrugineus*.

We also identified one gene in the *C. ferrugineus* transcriptome encoding the general odorant-binding protein (OBP), which was highly expressed in the gut of normally fed *C. ferrugineus* but was significantly down-regulated in starved beetles (Table S2). OBPs and CSPs have been reported to bind and transport lipophilic odorants, including sex pheromones, plant volatiles, and environmental odors [48]. OBPs are expressed in the sensilla of the antennae and in the ovipositor [49], supporting their functions as chemoreceptors of odors. They have also been reported to be a part of a feedback loop in moths’ pheromone glands, controlling the biosynthesis and release of sex pheromones [50].

The shuttle-craft gene may stimulate hormone production in the pheromone production of insects. Studies on shuttle-craft-like genes have focused on their role in the development of the central nervous system of *D. melanogaster* embryos [51], as well as their expression in the nervous and reproductive systems of adult flies [52]. Genes regulated by shuttle crafting have been reported to be down-regulated in males. It is interesting to note that these genes may be important in the development of neurons required for pheromone production in the cotton boll weevil [22].

Starvation-induced stress may also trigger a range of anti-starvation mechanisms. In addition to organismal systems, in general, the pathways identified in starving beetles can generally be categorized into two functions: metabolism and genetic information processing. Among these pathways, the spliceosome, amino acid–sugar and nucleotide–sugar metabolisms, fatty acid elongation, insulin-signaling pathway, ribosomes, and cytochrome P450 metabolism in response to xenobiotics have been found to be differentially expressed in starving beetles (Figure 4). During starvation in *C. ferrugineus*, carbohydrate metabolism emerged as one of the down-regulated pathways, with transcripts linked to chitinase (idgf), fructose-1,6-bisphosphatase (fbp), fructose-2,6-bisphosphatase (pfrx), and fructose-bisphosphate aldolase (ald1) genes being repressed (Table 2). Comparable findings were observed in *Lipaphis erysimi* under starvation conditions [53].

## 5. Conclusions

A comparative transcriptomics analysis of the guts of grain beetles under starvation or normal feeding conditions was conducted, providing a reference for future RNA-seq-based experiments. Genes differentially expressed between starved and normally fed beetles were identified through DEG analysis. Major changes in several metabolic pathways, including the JH pathway and the insulin pathway, were identified in grain beetles under starvation, warranting further studies. Our data shed light on the molecular mechanisms of grain beetles when faced with starvation. More importantly, our study lays the foundation for further revealing the molecular mechanisms of aggregation pheromone synthesis in rusty grain beetles.

## Figures and Tables

**Table 1:** Raw data of the six transcriptomes from starved and non-starved *C. ferrugineus*.

Sample Name	Reads	Bases (bp)	Q30 (bp)	N (%)	Q20 (%)	Q30 (%)
CK_1	51206932	7681039800	7040356784	0.001533	96.77	91.65
CK_2	48767240	7315086000	6687964471	0.001528	96.67	91.42
CK_3	53140036	7971005400	7286523463	0.001566	96.68	91.41
T_1	53257098	7988564700	7349637306	0.001544	96.92	92
T_2	55081590	8262238500	7586779831	0.001539	96.86	91.82
T_3	53413278	8011991700	7348302782	0.001534	96.78	91.71

**Table 2:** List of genes consistent with pheromone biosynthesis that were down-regulated in starved *C. ferrugineus* males compared with non-starved males.

Sequence Name	Description	Log_2_ Fold	Adjusted *p*-Value	Best Blast Hit
MVA Pathway				
DN8502	3-hydroxy-3-methylglutaryl coenzyme A synthase	0.48	0.041	gi|859132804|gb|AKO63317.1|3-hydroxy-3-methylglutaryl coenzyme A synthase (*Leptinotarsa decemlineata*)
DN33375	3-hydroxy-3-methylglutaryl-coenzyme A reductase	1.54	0.0058	gi|91086027|ref|XP_973160.1| PREDICTED: 3-hydroxy-3-methylglutaryl-coenzyme A reductase (*Tribolium castaneum*)
FAS Pathway				
DN26988	acetyl-CoA carboxylase	1.36	0.043	gi|919000753|ref|XP_013405631.1| PREDICTED: acetyl-CoA-carboxylase-like, partial (*Lingula anatina*)
DN2984	acetyl-CoA carboxylase	1.59	0.0055	gi|919033674|ref|XP_013400502.1| PREDICTED: acetyl-CoA-carboxylase-like isoform X1 (*Lingula anatina*)
DN24459	fatty acid synthase	1.23	0.032	gi|998254491|gb|AMK38868.1| fatty acid synthase 1 (*Colaphellus bowringi*)
DN14591	Z9 acyl-CoA desaturase	1.19	1.68 × 10^−6^	gi|302371202|ref|NP_001180578.1| Z9 acyl-CoA desaturase B (*Tribolium castaneum*)
DN666	fatty acyl-CoA reductase	1.11	9.02 × 10^−6^	gi|91084571|ref|XP_973790.1| PREDICTED: putative fatty acyl-CoA reductase CG5065 (*Tribolium castaneum*)
DN7491	alcohol dehydrogenase	1.40	0.018	gi|556772250|ref|XP_005981291.1| PREDICTED: zinc-type alcohol-dehydrogenase-like protein C1198.01-like (*Pantholops hodgsonii*)
DN59540	alcohol dehydrogenase (NADP(+))	2.13	6.60 × 10^−6^	gi|685824924|emb|CEF59978.1| alcohol dehydrogenase (NADP(+)) (*Strongyloides ratti*)
DN1181	acyl-CoA-binding protein	1.69	0.0035	gi|676433961|ref|XP_009047071.1| acyl-CoA-binding protein (*Lottia gigantea*)
DN13258	acetyl-CoA acetyltransferase	0.73	0.0058	gi|983657959|gb|AMB37467.1| acetyl-CoA acetyltransferase 2 (*Leptinotarsa decemlineata*)
DN3332	cytochrome P450 4ac1		8.32 × 10^−10^	sp|Q9VMS9|C4AC1_DROME probable cytochrome P450 4ac1 OS = *Drosophila melanogaster* OX = 7227 GN = Cyp4ac1 PE = 2 SV = 1
Juvenile Pathway				
DN5793	juvenile-hormone-binding protein	6.93	0.0025	gi|952530030|gb|KRT83356.1| hemolymph juvenile-hormone-binding protein, partial (*Oryctes borbonicus*)
Insulin-Signaling Pathway				
DN63954	putative sugar transporter 25	2.62	0.00082	gi|571330982|gb|AHF27423.1| putative sugar transporter 25 (*Phaedon cochleariae*)
DN14137	protein phosphatase-2C-containing protein	2.60	9.92 × 10^−5^	gi|170578383|ref|XP_001894385.1| protein phosphatase-2C-containing protein (*Brugia malayi*)
DN31519	insulin-degrading enzyme	2.45	4.95 × 10^−14^	gi|642914575|ref|XP_971897.2| PREDICTED: insulin-degrading enzyme (*Tribolium castaneum*)
DN21681	3-phosphoinositide-dependent protein kinase 1-like isoform X1	2.23	0.00080	gi|957826734|ref|XP_014663194.1| PREDICTED: 3-phosphoinositide-dependent protein kinase 1-like isoform X1 (*Priapulus caudatus*)
DN27944	insulin-degrading enzyme	2.21	5.25 × 10^−9^	gi|642914575|ref|XP_971897.2| PREDICTED: insulin-degrading enzyme (*Tribolium castaneum*)
DN63976	alternative oxidase, mitochondrial-like	2.24	0.0019	gi|340373435|ref|XP_003385247.1| PREDICTED: alternative oxidase, mitochondrial-like (*Amphimedon queenslandica*)
DN33569	serine/threonine-protein kinase Nek8	1.95	6.82 × 10^−5^	gi|405968936|gb|EKC33959.1| serine/threonine-protein kinase Nek8 (*Crassostrea gigas*)
Other Genes				
DN13679	protein shuttle craft	0.54	0.0023	gi|189237698|ref|XP_970597.2| PREDICTED: protein shuttle craft (*Tribolium castaneum*)
DN63354	ATP-citrate synthase	2.42	0.0023	gi|817189530|ref|XP_012270095.1| PREDICTED: ATP-citrate synthase (*Orussus abietinus*)
DN9662	glycerol-3-phosphate dehydrogenase	1.69	0.0053	gi|363736119|ref|XP_422110.3| PREDICTED: glycerol-3-phosphate dehydrogenase 1-like protein (*Gallus gallus*)
DN1017	6-phosphofructo-2-kinase/fructose-2,6-bisphosphatase 1	0.47	0.0096	gi|915659545|gb|KOC62482.1| 6-phosphofructo-2-kinase/fructose-2,6-bisphosphatase 1 (*Habropoda laboriosa*)
DN16578	fructose-bisphosphate aldolase-like	3.21	0.0093	gi|340385739|ref|XP_003391366.1| PREDICTED: fructose-bisphosphate aldolase-like (*Amphimedon queenslandica*)

## Data Availability

All the sequencing data generated in this study were submitted to the National Genomics Data Center under accession number: PRJNA948860.

## Supplementary Materials

The following supporting information can be downloaded at https://www.mdpi.com/article/10.3390/insects15020137/s1: Table S1. Primers design of selected genes for RT-qPCR; Table S2. Statistics of the overall sequences; Table S3. Annotations of the overall sequences; Table S4. The most significantly different expression pathway of the KEGG pathway analysis for starved vs. normally fed male beetles; Table S5. Information of top 10 up-regulated and down-regulated expressed genes (DEGs) in different comparisons; Table S6. All the putative genes in pheromone biosynthesis via the MVA pathway in *C. ferrugineus*; Table S7. All the putative genes in pheromone biosynthesis via the FAS pathway in *C. ferrugineus*.
