# Supplementary material for: Comparative Transcriptomics Analysis Reveals Rusty Grain Beetle’s Aggregation Pheromone Biosynthesis Mechanism in Response to Starvation
Source: Insects. 2024 Feb 19;15(2):137. doi: 10.3390/insects15020137 (PMC10888681; doi:10.3390/insects15020137)
Supplement: Supplementary file 1 [file insects-15-00137-s001.zip › insects-2847857-supplementary.pdf]

Table S1. Primers design of selected genes for RT-qPCR.

Genes	Primer Sequence	
ARF1	Forward: GATGCCGTCCTGTTGATTTT	Reverse: GGCCCAGCTTGTCTGTTATC
HMGR	Forward: TCGCCTGCTTGTCGGTTAT	Reverse: CATACCTGTGTGCGTGGAC
Desaturase	Forward:TGCTTATTTGCACGCCATCG	Reverse:TCGGTCTCGCTGTACTTGTG
FPPS	Forward:TACCCGGCAAAAATGTTCGT	Reverse: CCTTGGCCACGATATAACTCC

Note: The primer sequences of ARF1 were followed by [32].

Table S2. Statistics of the overall sequences

Categories	Transcript	Unigene
Sequence Number	125338	42389
Max. Length (bp)	32626	32626
Mean Length (bp)	1558.19	1233.27
N50 (bp)	2458	2212
N50 Sequence No.	22645	6340
N90 (bp)	657	449
N90 Sequence No.	81323	28021
GC%	36.33	35.90

Table S3. Annotations of the overall sequences

Database Number Percentage	Database Percentage	Number	Database Number Percentage
NR	16364		38.60
GO	12078		28.49
KEGG	9570		22.58
Pfam	11649		27.48
eggNOG	15674		36.98
Swissprot	12730		30.03
In all database	6474		15.27

Table S4. The most significantly different expression pathway of the KEGG pathway analysis for starved vs normally fed male beetles.

Pathway ID	Pathway	DEG_number	total_number	*p*-Value	FDR
ko03040	Spliceosome	60	207	6.37×10^{-10}	9.91× 10−08
ko00520	Amino sugar and nucleotide sugar	39	107	5.06×10^{-10}	9.91× 10−08

	metabolism				
ko00500	Starch and sucrose metabolism	25	69	7.44×10^{-07}	7.72× 10−05
ko03050	Proteasome	23	69	1.04×10^{-05}	0.00081
ko03008	Ribosome biogenesis in eukaryotes	33	121	1.89×10^{-05}	0.0012
ko03430	Mismatch repair	13	30	3.95×10^{-05}	0.0020
ko03030	DNA replication	18	53	7.10×10^{-05}	0.0032
ko04910	Insulin signaling pathway	39	167	0.00015	0.0060
ko05134	Legionellosis	20	67	0.00022	0.0076
ko00604	Glycosphingolipid biosynthesis - ganglio series	6	9	0.00028	0.0087
ko04612	Antigen processing and presentation	25	95	0.00034	0.0097
ko04141	Protein processing in endoplasmic reticulum	44	209	0.00069	0.017
ko00052	Galactose metabolism	18	62	0.00065	0.017
ko04213	Longevity regulating pathway - multiple species	23	91	0.0011	0.024
ko00603	Glycosphingolipid biosynthesis - globo and isoglobo series	6	11	0.0012	0.025

Table S5. Information of top 10 up-regulated and down-regulated expressed genes (DEGs) in different comparisons.

Gene ID	Description	log_2 Fold Change	adjustedp-Value	Best blast Hit
DN36036	Prostatic acid phosphatase	−5.80	0.0010	Tribolium castaneum
DN14259	salivary protein	−2.59	1.75×10^{-07}	Phlebotomus ariasi
DN63954	putative sugar transporter 25	−2.56	0.00082	Phaedon cochleariae
DN14137	Protein phosphatase 2C containing protein	−2.55	9.92×10^{-05}	Brugia malayi
DN31519	insulin-degrading enzyme	−2.38	4.95×10^{-14}	Tribolium castaneum
DN21681	3-phosphoinositide-dependent protein kinase 1-like isoform X1	−2.25	0.00080	Priapulus caudatus
DN27944	insulin-degrading enzyme	−2.21	5.25×10^{-09}	Tribolium castaneum
DN63976	alternative oxidase, mitochondrial-like	−2.19	0.0019	Amphimedon queenslandica
DN59540	Alcohol dehydrogenase [NADP(+)]	−2.06	6.60×10^{-06}	Strongyloides ratti
DN33569	Serine/threonine-protein kinase Nek8	−1.91	0.0010	Crassostrea gigas
DN9923	glycoside hydrolase family 1	5.90	4.72×10^{-06}	Phyllotreta striolata
DN8951	cytochrome P450 monooxygenase	4.89	0.00025	Tribolium castaneum
DN26488	glutamate receptor ionotropic, kainate 2 isoform X6	4.37	0.023	Tribolium castaneum
DN3904	chitinase	4.27	2.17×10^{-58}	Monochamus alternatus
DN14148	transmembrane protease serine 9	4.22	0.030	Tribolium castaneum
DN11370	Potassium channel subfamily T member 1-like Protein	4.18	0.021	Tribolium castaneum
DN13853	vitellogenin receptor	4.08	0.042	Tribolium

				castaneum
DN812	Lipase 3	3.83	1.55×10^{-29}	Zootermopsis nevadensis
DN12993	cytochrome P450 6bq17	3.72	0.013	Leptinotarsa decemlineata
DN39974	general odorant-binding protein 72	3.76	0.012	Tribolium castaneum

Table S6. All the putative genes in pheromone biosynthesis via the MVA pathway in *C. ferrugineus*

Sequence Name	Length(bp)	Description	Best blast Hit
DN8502	2684	3-hydroxy-3-methylglutaryl coenzyme A synthase	gi|859132804|gb|AKO63317.1| 3-hydroxy-3-methylglutaryl coenzyme A synthase (*Leptinotarsa decemlineata*)
DN14	3765	3-hydroxy-3-methylglutaryl coenzyme A reductase 2	gi|859132811|gb|AKO63319.1| 3-hydroxy-3-methylglutaryl coenzyme A reductase 2 (*Leptinotarsa decemlineata*)
DN13258	2520	acetyl-CoA acetyltransferase 2	gi|983657959|gb|AMB37467.1| acetyl-CoA acetyltransferase 2 (*Leptinotarsa decemlineata*)
DN7232	1958	acetyl-CoA acetyltransferase, mitochondrial isoform X1	gi|189234785|ref|XP_975008.2| PREDICTED: acetyl-CoA acetyltransferase, mitochondrial isoform X1 (*Tribolium castaneum*)
DN4867	1986	mevalonate kinase	gi|859132814|gb|AKO63320.1| mevalonate kinase (*Leptinotarsa decemlineata*)
DN1057	1828	diphosphomevalonate decarboxylase	gi|385200014|gb|AFI45055.1| diphosphomevalonate decarboxylase (*Dendroctonus ponderosae*)
DN14040	2079	geranylgeranyl diphosphate synthase	gi|549439117|gb|AGX25357.1| geranylgeranyl diphosphate synthase (*Pissodes strobi*)
DN43621	412	isopentenyl diphosphate synthase	gi|943360326|gb|ALL35402.1| isopentenyl diphosphate synthase (*Psylliodes chrysocephalus*)
DN34563	1439	isoprenyl diphosphate synthase	gi|449139004|gb|AGE89831.1| isoprenyl diphosphate synthase

			(*Phaedon cochleariae*)
DN17749	1283	isopentenyl diphosphate synthase	gi|943360322|gb|ALL35400.1| isopentenyl diphosphate synthase (*Phyllotreta striolata*)
DN17069	854	farnesyl pyrophosphate synthase	gi|282158091|ref|NP_001164089.1| farnesyl pyrophosphate synthase (*Tribolium castaneum*)
DN3052	2122	farnesyl pyrophosphate synthase	gi|1008444024|ref|XP_015836774.1 | PREDICTED: farnesyl pyrophosphate synthase isoform X1 (*Tribolium castaneum*)
DN34020	437	cytochrome P450-4c	gi|936578848|ref|XP_014204067.1| PREDICTED: cytochrome P450 4C1-like (*Copidosoma floridanum*)
DN57140	387	Cytochrome P450-6a	gi|1004399086|gb|KYB27039.1| Cytochrome P450 6a2-like Protein (*Tribolium castaneum*)
DN11348	539	Cytochrome P450-6a	gi|91084707|ref|XP_969633.1| PREDICTED: probable cytochrome P450 6a23 (*Tribolium castaneum*)
DN9170	1751	cytochrome P450-4c	gi|91094839|ref|XP_971612.1| PREDICTED: cytochrome P450 4C1 (*Tribolium castaneum*)
DN1125	319	cytochrome P450 9e	gi|91088761|ref|XP_975385.1| PREDICTED: cytochrome P450 9e2 (*Tribolium castaneum*)
DN25899	463	cytochrome P450 9e	gi|1008450276|ref|XP_015838490.1 | PREDICTED: cytochrome P450 9e2-like (*Tribolium castaneum*)
DN14817	414	cytochrome P450 6b	gi|478734979|gb|AGJ51945.1| cytochrome P450 CYP6BQ22 (*Dastarcus helophoroides*)

Table S7. All the putative genes in pheromone biosynthesis via the FAS pathway in *C. ferrugineus*

Sequence Name	Length	Description	Best blast Hit
DN26988	5727	acetyl-CoA carboxylase	gi|919000753|ref|XP_013405631.1| PREDICTED: acetyl-CoA carboxylase-like, partial (*Lingula anatina*)
DN2984	5449	acetyl-CoA carboxylase	gi|919033674|ref|XP_013400502.1| PREDICTED: acetyl-CoA carboxylase-like isoform X1 (*Lingula*

			anatina)
DN4246	8236	acetyl-CoA carboxylase	gi|642926044|ref|XP_008194742.1| PREDICTED: acetyl-CoA carboxylase isoform X4 (*Tribolium castaneum*)
DN451	7614	fatty acid synthase	gi|998254491|gb|AMK38868.1| fatty acid synthase 1 (*Colaphellus bowringi*)
DN451	448	fatty acid synthase	gi|478250393|gb|ENN70888.1| hypothetical protein YQE_12293, partial (*Dendroctonus ponderosae*)
DN5055	7842	fatty acid synthase	gi|546672885|gb|ERL84608.1| hypothetical protein D910_02036 (*Dendroctonus ponderosae*)
DN13200	2686	fatty acid synthase	gi|936579093|ref|XP_014204155.1| PREDICTED: fatty acid synthase-like isoform X1 (*Copidosoma floridanum*)
DN13306	898	fatty acid synthase	gi|478252126|gb|ENN72557.1| hypothetical protein YQE_10897, partial (*Dendroctonus ponderosae*)
DN13809	4400	fatty acid synthase	gi|833654169|gb|AKM28424.1| fatty acid synthase 2 (*Aphis gossypii*)
DN14149	720	fatty acid synthase	gi|478250393|gb|ENN70888.1| hypothetical protein YQE_12293, partial (*Dendroctonus ponderosae*)
DN14585	637	fatty acid synthase	gi|808127224|ref|XP_012166572.1| PREDICTED: LOW QUALITY PROTEIN: fatty acid synthase (*Bombus terrestris*)
DN17237	853	fatty acid synthase	gi|350407733|ref|XP_003488176.1| PREDICTED: fatty acid synthase (*Bombus impatiens*)
DN20777	1243	fatty acid synthase	gi|1008441829|ref|XP_015836196.1| PREDICTED: fatty acid synthase (*Tribolium castaneum*)
DN2296	6583	fatty acid synthase	gi|642914795|ref|XP_008190356.1| PREDICTED: fatty acid synthase (*Tribolium castaneum*)
DN24241	430	fatty acid synthase	gi|827552646|ref|XP_012548082.1| PREDICTED: LOW QUALITY PROTEIN: p270 isoform X1 (*Bombyx mori*)
DN24375	356	fatty acid synthase	gi|939666222|ref|XP_014280344.1| PREDICTED: fatty acid synthase-like

			(*Halyomorpha halys*)
DN24459	327	fatty acid synthase	gi|998254491|gb|AMK38868.1| fatty acid synthase 1 (*Colaphellus bowringi*)
DN26951	397	fatty acid synthase	gi|970895185|ref|XP_015113057.1| PREDICTED: fatty acid synthase-like (*Diachasma alloeum*)
DN27948	1080	fatty acid synthase	gi|478250393|gb|ENN70888.1| hypothetical protein YQE_12293, partial (*Dendroctonus ponderosae*)
DN3683	8015	fatty acid synthase	gi|642910328|ref|XP_008200285.1| PREDICTED: fatty acid synthase (*Tribolium castaneum*)
DN37960	1788	fatty acid synthase	gi|972194346|ref|XP_015179002.1| PREDICTED: fatty acid synthase (*Polistes dominula*)
DN38748	329	fatty acid synthase	gi|998254491|gb|AMK38868.1| fatty acid synthase 1 (*Colaphellus bowringi*)
fatty acid transport protein			
DN805	2935	fatty acid transport protein	gi|642923280|ref|XP_008193688.1| PREDICTED: long-chain fatty acid transport protein 4 isoform X1 (*Tribolium castaneum*)
DN18413	396	fatty acid transport protein	gi|642923280|ref|XP_008193688.1| PREDICTED: long-chain fatty acid transport protein 4 isoform X1 (Tribolium castaneum)
DN2523	2017	fatty acid transport protein	gi|91086105|ref|XP_967675.1| PREDICTED: long-chain fatty acid transport protein 4 (*Tribolium castaneum*)
Acyl-CoA dehydrogenase			
DN2581	3478	Acyl-CoA dehydrogenase	gi|642938637|ref|XP_008197587.1| PREDICTED: short/branched chain specific acyl-CoA dehydrogenase, mitochondrial (*Tribolium castaneum*)
DN348	1959	Acyl-CoA dehydrogenase	gi|91079744|ref|XP_970506.1| PREDICTED: probable medium-chain specific acyl-CoA dehydrogenase, mitochondrial (*Tribolium castaneum*)
DN5059	2150	Acyl-CoA dehydrogena	gi|642915063|ref|XP_008190394.1| PREDICTED: acyl-CoA

		se	dehydrogenase family member 9, mitochondrial (*Tribolium castaneum*)
DN811	2224	Acyl-CoA dehydrogenase	gi|91088951|ref|XP_973830.1| PREDICTED: short-chain specific acyl-CoA dehydrogenase, mitochondrial (*Tribolium castaneum*)
DN9993	534	Acyl-CoA dehydrogenase	gi|662190906|ref|XP_008468241.1| PREDICTED: probable medium-chain specific acyl-CoA dehydrogenase, mitochondrial (*Diaphorina citri*)
DN10841	2696	Acyl-CoA dehydrogenase	gi|91076006|ref|XP_966406.1| PREDICTED: very long-chain specific acyl-CoA dehydrogenase, mitochondrial (*Tribolium castaneum*)
DN1273	1691	Acyl-CoA dehydrogenase	gi|91079744|ref|XP_970506.1| PREDICTED: probable medium-chain specific acyl-CoA dehydrogenase, mitochondrial (*Tribolium castaneum*)
DN13359	326	Acyl-CoA dehydrogenase	gi|642938637|ref|XP_008197587.1| PREDICTED: short/branched chain specific acyl-CoA dehydrogenase, mitochondrial (*Tribolium castaneum*)
DN9959	2483	3-ketoacyl-CoA thiolase	gi|91087491|ref|XP_968373.1| PREDICTED: 3-ketoacyl-CoA thiolase, mitochondrial (*Tribolium castaneum*)
DN16536	474	3-ketoacyl-CoA thiolase	gi|663287133|ref|XP_008500452.1| PREDICTED: 3-ketoacyl-CoA thiolase, peroxisomal (*Calypte anna*)
3-hydroxyacyl-CoA dehydrogenase			
DN164	3279	3-hydroxyacyl-CoA dehydrogenase	gi|91078584|ref|XP_971954.1| PREDICTED: 3-hydroxyacyl-CoA dehydrogenase type-2 (*Tribolium castaneum*)
enoyl-CoA hydratase			
DN3785	1584	3-hydroxyacyl-CoA dehydrogena	gi|91076844|ref|XP_974775.1| PREDICTED: probable enoyl-CoA hydratase (*Tribolium castaneum*)

		se	
DN10753	1604	Desaturase	gi|328722879|ref|XP_003247697.1| PREDICTED: acyl-CoA Delta (11) desaturase isoform X1 (*Acyrthosiphon pisum*)
DN14591	2190	Desaturase	gi|302371202|ref|NP_001180578.1| Z9 acyl-CoA desaturase B (*Tribolium castaneum*)
DN35858	786	Desaturase	gi|768419096|ref|XP_011550250.1| PREDICTED: stearoyl-CoA desaturase 5-like (*Plutella xylostella*)
DN4192	2745	Desaturase	gi|984880662|ref|NP_001306191.1| acyl-CoA Delta(11) desaturase (*Tribolium castaneum*)
DN6011	2756	Desaturase	gi|907678927|ref|XP_013106080.1| PREDICTED: acyl-CoA Delta(11) desaturase (*Stomoxys calcitrans*)
DN7337	1764	Desaturase	gi|1000734683|ref|XP_015589799.1| PREDICTED: acyl-CoA Delta(11) desaturase isoform X1 (*Cephus cinctus*)
DN748	2342	Desaturase	gi|984880672|ref|NP_001306192.1| acyl-CoA Delta(11) desaturase-like (*Tribolium castaneum*)
DN1161	2785	Fatty acyl-CoA reductase	gi|642916861|ref|XP_008199532.1| PREDICTED: putative fatty acyl-CoA reductase CG5065 isoform X2 (*Tribolium castaneum*)
DN12539	2315	Fatty acyl-CoA reductase	gi|1008443007|ref|XP_008194927.2| PREDICTED: fatty acyl-CoA reductase 1 isoform X1 (*Tribolium castaneum*)
DN12880	1378	Fatty acyl-CoA reductase	gi|189239820|ref|XP_971534.2| PREDICTED: putative fatty acyl-CoA reductase CG5065 (*Tribolium castaneum*)
DN12939	2465	Fatty acyl-CoA reductase	gi|985386804|ref|XP_015371677.1| PREDICTED: putative fatty acyl-CoA reductase CG5065 (*Diuraphis noxia*)
DN17133	303	Fatty acyl-CoA	gi|939637560|ref|XP_014294630.1| PREDICTED: putative fatty acyl-

		reductase	CoA reductase CG5065, partial (*Halyomorpha halys*)
DN18398	850	Fatty acyl-CoA reductase	gi|91087837|ref|XP_967757.1| PREDICTED: putative fatty acyl-CoA reductase CG5065 (*Tribolium castaneum*)
DN18398	935	Fatty acyl-CoA reductase	gi|817187712|ref|XP_012288416.1| PREDICTED: fatty acyl-CoA reductase 1 (*Orussus abietinus*)
DN2670	2084	Fatty acyl-CoA reductase	gi|91085011|ref|XP_973467.1| PREDICTED: putative fatty acyl-CoA reductase CG8306 (*Tribolium castaneum*)
DN33105	2209	Fatty acyl-CoA reductase	gi|1008441937|ref|XP_015836217.1| PREDICTED: fatty acyl-CoA reductase 1 isoform X2 (*Tribolium castaneum*)
DN36061	884	Fatty acyl-CoA reductase	gi|951561237|ref|XP_014479570.1| PREDICTED: putative fatty acyl-CoA reductase CG5065 (*Dinoponera quadriceps*)
DN4075	2799	Fatty acyl-CoA reductase	gi|189238048|ref|XP_001811309.1| PREDICTED: putative fatty acyl-CoA reductase CG5065 (*Tribolium castaneum*)
DN4372	2406	Fatty acyl-CoA reductase	gi|189239820|ref|XP_971534.2| PREDICTED: putative fatty acyl-CoA reductase CG5065 (*Tribolium castaneum*)
DN4479	1174	Fatty acyl-CoA reductase	gi|659495078|gb|AID66650.1| fatty acyl reductase (*Agrotis segetum*)
DN4898	2435	Fatty acyl-CoA reductase	gi|270008526|gb|EFA04974.1| Putative fatty acyl-CoA reductase CG5065-like Protein (*Tribolium castaneum*)
DN50015	389	Fatty acyl-CoA reductase	gi|985386804|ref|XP_015371677.1| PREDICTED: putative fatty acyl-CoA reductase CG5065 (*Diuraphis noxia*)
DN510	1975	Fatty acyl-CoA reductase	gi|572270973|ref|XP_006613464.1| PREDICTED: putative fatty acyl-CoA reductase CG5065-like (*Apis dorsata*)

DN6636	2182	Fatty acyl-CoA reductase	gi|91084843|ref|XP_966905.1| PREDICTED: putative fatty acyl-CoA reductase CG5065 (*Tribolium castaneum*)
DN666	3915	Fatty acyl-CoA reductase	gi|91084571|ref|XP_973790.1| PREDICTED: putative fatty acyl-CoA reductase CG5065 (*Tribolium castaneum*)
DN7489	2261	Fatty acyl-CoA reductase	gi|189239820|ref|XP_971534.2| PREDICTED: putative fatty acyl-CoA reductase CG5065 (*Tribolium castaneum*)
DN9924	1623	Fatty acyl-CoA reductase	gi|91087837|ref|XP_967757.1| PREDICTED: putative fatty acyl-CoA reductase CG5065 (*Tribolium castaneum*)
DN5736	1725	acyl-CoA-binding protein	gi|91090704|ref|XP_974824.1| PREDICTED: putative acyl-CoA-binding protein (*Tribolium castaneum*)
DN1181	5019	acyl-CoA-binding protein	gi|676433961|ref|XP_009047071.1| hypothetical protein LOTGIDRAFT_205113 (*Lottia gigantea*)
DN13258	2520	acetyl-CoA acetyltransferase(ACAT)	gi|983657959|gb|AMB37467.1| acetyl-CoA acetyltransferase 2 (*Leptinotarsa decemlineata*)
DN7232	1958	acetyl-CoA acetyltransferase(ACAT)	gi|189234785|ref|XP_975008.2| PREDICTED: acetyl-CoA acetyltransferase, mitochondrial isoform X1 (*Tribolium castaneum*)

Reference

1. Zhang, M.; Du, M.-Y.; Wang, G.-X.; Wang, Z.-Y.; Lu, Y.-J. Identification, mRNA expression, and functional analysis of chitin synthase 2 gene in the rusty grain beetle, *Cryptolestes ferrugineus*. *J. Stored Prod. Res.* **2020**, *87*, 101622. https://doi.org/10.1016/j.jspr.2020.101622.
